# The Impact of the COVID-19 Pandemic on Head and Neck Cancer Diagnosis in the Piedmont Region, Italy: Interrupted Time-Series Analysis

**DOI:** 10.3389/fpubh.2022.809283

**Published:** 2022-02-21

**Authors:** Maja Popovic, Valentina Fiano, Giovenale Moirano, Luigi Chiusa, David I. Conway, Paolo Garzino Demo, Marco Gilardetti, Giuseppe Carlo Iorio, Chiara Moccia, Oliviero Ostellino, Giancarlo Pecorari, Guglielmo Ramieri, Umberto Ricardi, Giuseppe Riva, Shama Virani, Lorenzo Richiardi

**Affiliations:** ^1^Cancer Epidemiology Unit, Department of Medical Sciences, University of Turin, Turin, Italy; ^2^SC Epidemiologia dei Tumori CRPT U, Azienda Ospedaliero-Universitaria (A.O.U.) Città della Salute e della Scienza di Torino, Centro di Riferimento per l'Epidemiologia e la Prevenzione Oncologica (CPO) Piemonte, Turin, Italy; ^3^Pathology Unit, Azienda Ospedaliero-Universitaria (A.O.U.) Città della Salute e della Scienza Hospital, Turin, Italy; ^4^School of Medicine, Dentistry and Nursing, University of Glasgow, Glasgow, United Kingdom; ^5^Maxillofacial Unit, Azienda Ospedaliero-Universitaria (A.O.U.) Città della Salute e della Scienza Hospital, Turin, Italy; ^6^Department of Oncology, University of Turin, Turin, Italy; ^7^Oncology Unit, Azienda Ospedaliero-Universitaria (A.O.U.) Città della Salute e della Scienza Hospital, Turin, Italy; ^8^Division of Otorhinolaryngology, Department of Surgical Sciences, University of Turin, Turin, Italy; ^9^Genomic Epidemiology Branch, International Agency for Research on Cancer (IARC/WHO), Lyon, France

**Keywords:** COVID-19, head and neck, cancer detection, Italy, interrupted time-series analysis (ITSA), time trend analyses, head and neck cancers, cancer

## Abstract

**Background:**

The COVID-19 pandemic has likely affected the most vulnerable groups of patients and those requiring time-critical access to healthcare services, such as patients with cancer. The aim of this study was to use time trend data to assess the impact of COVID-19 on timely diagnosis and treatment of head and neck cancer (HNC) in the Italian Piedmont region.

**Methods:**

This study was based on two different data sources. First, regional hospital discharge register data were used to identify incident HNC in patients ≥18 years old during the period from January 1, 2015, to December 31, 2020. Interrupted time-series analysis was used to model the long-time trends in monthly incident HNC before COVID-19 while accounting for holiday-related seasonal fluctuations in the HNC admissions. Second, in a population of incident HNC patients eligible for recruitment in an ongoing clinical cohort study (HEADSpAcE) that started before the COVID-19 pandemic, we compared the distribution of early-stage and late-stage diagnoses between the pre-COVID-19 and the COVID-19 period.

**Results:**

There were 4,811 incident HNC admissions in the 5-year period before the COVID-19 outbreak and 832 admissions in 2020, of which 689 occurred after the COVID-19 outbreak in Italy. An initial reduction of 28% in admissions during the first wave of the COVID-19 pandemic (RR 0.72, 95% CI 0.62–0.84) was largely addressed by the end of 2020 (RR 0.96, 95% CI 0.89–1.03) when considering the whole population, although there were some heterogeneities. The gap between observed and expected admissions was particularly evident and had not completely recovered by the end of the year in older (≥75 years) patients (RR: 0.88, 0.76–1.01), patients with a Romano-Charlson comorbidity index below 2 (RR 0.91, 95% CI: 0.84–1.00), and primary surgically treated patients (RR 0.88, 95% CI 0.80–0.97). In the subgroup of patients eligible for the ongoing active recruitment, we observed no evidence of a shift toward a more advanced stage at diagnosis in the periods following the first pandemic wave.

**Conclusions:**

The COVID-19 pandemic has affected differentially the management of certain groups of incident HNC patients, with more pronounced impact on older patients, those treated primarily surgically, and those with less comorbidities. The missed and delayed diagnoses may translate into worser oncological outcomes in these patients.

## Introduction

Since the outbreak of coronavirus disease 2019 (COVID-19) pandemic important adjustments have been introduced in the healthcare settings to avoid hospital outbreaks and ensure necessary resources for COVID-19 patients. These included partial or complete interruption of diagnostic workflows, elective treatments, and management of routine follow-ups (1). Although those policies were targeted mainly to more deferrable activities, the COVID-19-related containment strategies are likely to have also affected the most vulnerable groups of patients and those requiring time-critical access to healthcare service, such as patients with cancer.

COVID-19 has triggered a decline in referral, diagnosis and treatment for cancer patients all over the world (2–5), raising concerns about a substantial increase in the number of avoidable cancer deaths in the near future (6). Nationally and locally imposed restrictions, guidelines for management of patients during different pandemic waves, changes in health-seeking behavior and the perception that hospitals are high-risk areas in terms of COVID-19 transmission risk, are all likely to have contributed to the observed reductions in the number of newly diagnosed cancer cases during the pandemic (7).

Head and neck cancers (HNC) are characterized by relatively fast growth, and timely diagnosis with prompt treatment initiation are essential for optimal oncologic outcomes in these patients (8). The effects of delayed HNC diagnosis and treatment are both immediate and long-term, and are expected to result in more advanced stages at presentation, an increased risk of relapse, worse treatment response and decreased short- and long-term survival (9). Patients with HNC are often diagnosed at an advanced stage, with previously reported prevalence of stage III-IV disease of 55 and 60% within the European ARCAGE and the UK HN5000 study, respectively (10, 11). Delays in the diagnosis and treatment of HNC due to the COVID-19 pandemic have been reported in several countries (12–16), causing more admissions in advanced stage disease and an increase in more complex reconstructive procedures.

Assessing the impact of COVID-19 on timely HNC diagnosis and treatment is complex, and the analyses must consider underlying temporal trends in admissions. Moreover, it is necessary to wait long enough to evaluate whether the COVID-19 impact has been limited to the first pandemic wave only or has lasting effects. Addressing the delayed diagnoses and treatment backlogs caused by the pandemic may take significant time to return to the pre-pandemic levels, contributing further to the existing healthcare crisis and increasing the number of potentially avoidable deaths due to cancer.

This study aimed at evaluating the temporal trends in HNC detection in the Piedmont Region, Italy, before and during the COVID-19 pandemic to indirectly assess the impact of COVID-19 on timely HNC diagnosis and treatment. Taking advantage of an ongoing HEADSpAcE study (17) recruitment, which had started in one of the largest tertiary care hospitals in Piedmont before the COVID-19 pandemic, we analyzed the stage at diagnosis of incident HNC in the pre-COVID-19 period of recruitment and in the periods following the COVID-19 outbreak.

## Methods

### Study Population

This study was based on two different data sources. First, to identify potential variations in the temporal trends in HNC detection we retrieved data from the Hospital Information System (HIS), which includes all hospital discharge records (HDRs) from public and private hospitals in Piedmont (4,311,217 inhabitants as of January 1, 2020, corresponding to 7.2% of the national population). The HIS routinely collects both inpatient and day-case hospital activity from all regional private and public hospitals funded by the Italian National Health Service (NHS). The NHS covers the entire population and private funding is residual in the case of severe diseases like cancer.

To avoid potentially different referral practices during the pandemic, we considered only patients with both residence and hospitalization in Piedmont. Among all patients ≥18 years of age discharged between January 1, 2015 and December 31, 2020, we selected those with the following International Classification of Diseases, 9th Revision, Clinical Modification (ICD-9-CM) codes (included as primary or any of the five secondary diagnoses reported in HDR): 140.0–149 (malignant neoplasm of lip, oral cavity, and pharynx), 150.0 (malignant neoplasm of cervical esophagus), 161.0–161.9 (malignant neoplasm of larynx) and 195.0 (malignant neoplasm of head, face and neck). Of this cohort, patients with potentially prevalent cancer were excluded, i.e., patients who had been hospitalized with any of the above listed HNC diagnoses at any time during the 10 years prior to the index HNC hospitalization.

Data were extracted as anonymous individual records, and included age, gender, region of residence, date of admission, length of hospital stay, primary and up to five secondary diagnoses (ICD-9-CM codes), type of admission [surgical vs. non-surgical, based on the surgical procedures from the diagnosis-related group (DRG) claims] and date of discharge. Disease stage is not routinely collected through HDRs and was not available for the analyses based on these data. The Charlson Comorbidity Index (CCI), as adapted by Romano et al. (18), was computed considering all hospital discharges that occurred within 12 months before the index admission. We considered all patients who had no surgical DRG claim within the 6 months from the index admission (including the index admission) as non-surgically treated patients.

Second, for the analysis of temporal trends in the disease stage at diagnosis we analyzed clinical data of patients eligible for an ongoing recruitment at the University Hospital “Città della Salute e della Scienza di Torino”. This recruitment is being conducted within the context of the HEADSpAcE study, an EU-funded project that brings together a consortium of 15 institutions with an aim, among others, to elucidate reasons for late diagnosis of HNC (17). The following diagnoses are eligible [International classification of disease for oncology, Third Edition (ICD-0-3) topographic codes]: C00-C14 (lip, oral cavity and pharynx; excluding external lip C00.0-C00.2, parotid gland C07, other and unspecified major salivary glands C08, and nasopharynx C11) and C32 (larynx). The ongoing recruitment started on September 1, 2019, at the two HNC surgery departments (Otorhinolaryngology and Maxillofacial Surgery) where patients with HNC or suspected HNC are being referred. For the eligible patients, the following data were extracted from the electronic medical records: age, sex, date of diagnosis, tumor site (ICD-0-3 codes). Disease stage at diagnosis (TNM Classification of Malignant Tumors, AJCC cancer staging manual, VIII ed) was systematically reviewed for each patient based on the available pathological and clinical information. The patients were grouped into early-stage (I and II) and late-stage (III and IV) at diagnosis.

The HEADSpAcE study was approved by the local Ethical Committee (Protocol Number CS2/1354) and all the participating patients gave their written consent at enrolment.

### Statistical Analyses

**Figure 1:**
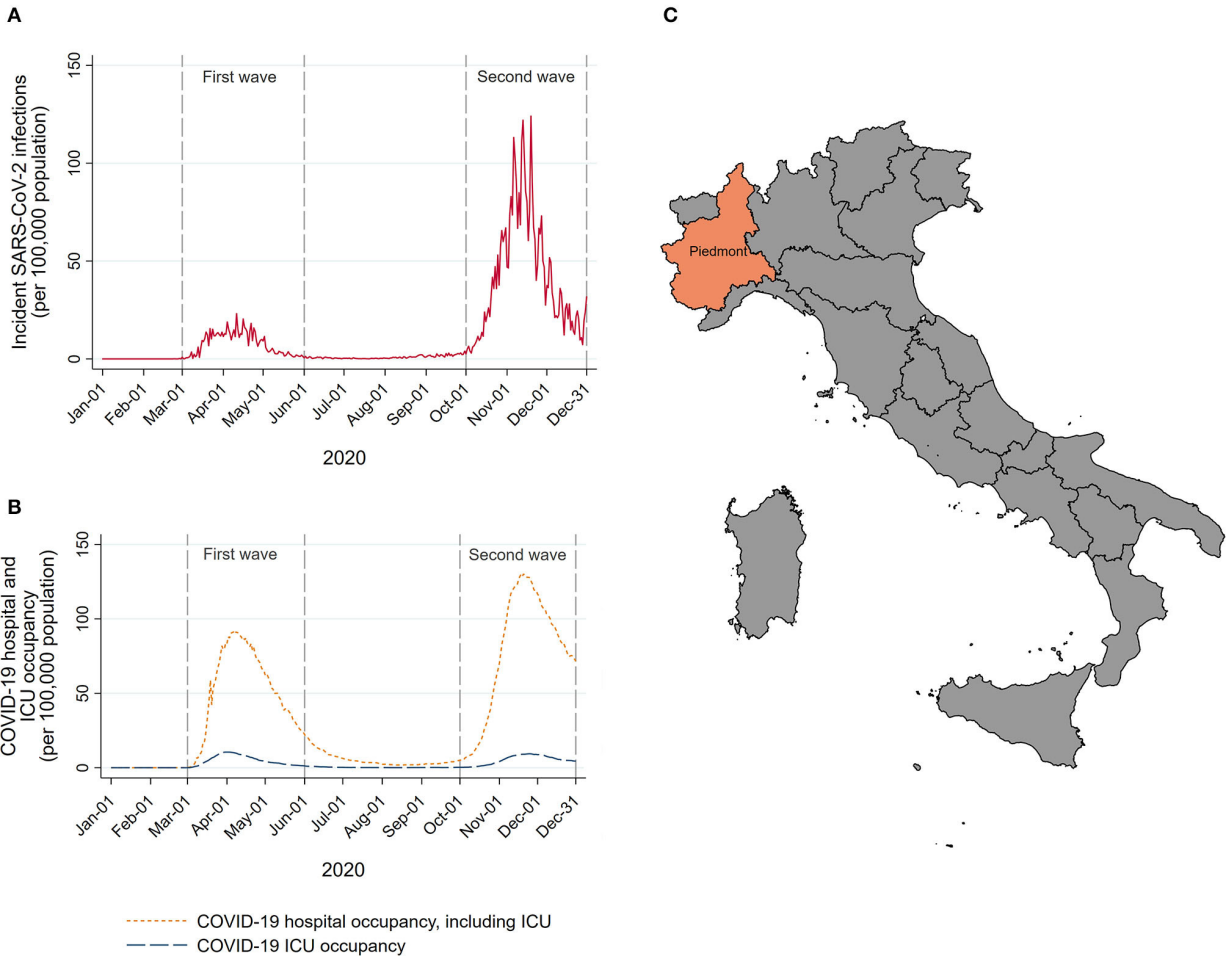
Number of new SARS-CoV-2 infections **(A)**, and COVID-19 hospital and intensive care unit (ICU) occupancy **(B)** per 1,00,000 resident population in Piedmont during 2020. A map of Italy with Piedmont location **(C)**.

The interrupted time-series analysis was used to model the trends in monthly incident HNC before COVID-19 accounting for seasonal and long-term time trends. Given the distribution of the daily number of COVID-19 cases in this region (Figure 1, data obtained from the national surveillance system available at the website of the Italian Ministry of Health/Civil Protection Department) (19) and consequent introduction, at the beginning of March 2020, of governmental COVID-19-related containment strategies at national level, we considered March 1, 2020, as the start of the COVID-19 pandemic in Piedmont. Data from all months starting from January 01, 2015 until February 29, 2020 were included in the pre-COVID-19 period, while all the data from the remaining months until December 31, 2020 were included in the COVID-19 period. Models were fitted using a negative binomial regression. We included a linear term for time to model the underlying long-term trend between 2015 and 2020, and Fourier terms, consisting of two sine/cosine pairs to model seasonal holiday-related fluctuations in the HNC admissions. This model was used to predict what the trend in HNC from March 2020 would have been had COVID-19 not occurred, calculating the expected number of HNCs. The expected number was then compared with the observed number of HNCs in the same period by calculating the rate ratios (RRs) and the corresponding 95% confidence intervals (CIs) using the Byar's approximation (20). These analyses were further stratified by the age at diagnosis (<65, 65–75, and ≥75 years of age), tumor subsite (oral cavity, oropharynx, larynx, other HNC subsites), CCI (<2 and ≥2) and type of treatment (surgical and non-surgical treatments, including palliative and no treatment at all). As one does not expect clinical or biological seasonal effects in HNC occurrence (any seasonal variations are likely to capture lower diagnostic intensity during holidays), we performed sensitivity analysis for the main non-stratified model by including linear term for long-term time trend only (without modeling seasonal trends).

To compare the disease stage distribution, the percentage of late-stage HNC diagnoses during the pre-pandemic period of the HEADSpAcE recruitment (September 1, 2019–February 29, 2020) was compared, using the chi-squared test, with the percentage of late-stage diagnoses during the periods following the COVID-19 outbreak: (i) the first pandemic wave (March 1, 2020–May 31, 2020), (ii) re-opening period (June 1, 2020–September 30, 2020), and (iii) the second pandemic wave (October 1, 2020–December 31, 2020). The analyzed periods were defined a priori based on the pandemic dynamics and the number of new confirmed COVID-19 cases in Piedmont (Figure 1).

Data management was performed using SAS™ engine V9.4 (SAS Institute Inc., Cary, NC, USA), while the statistical analyses were performed using STATA version 15 (StataCorp, College Station, TX, USA).

## Results

### Interrupted Time-Series Analyses

**Figure 2:**
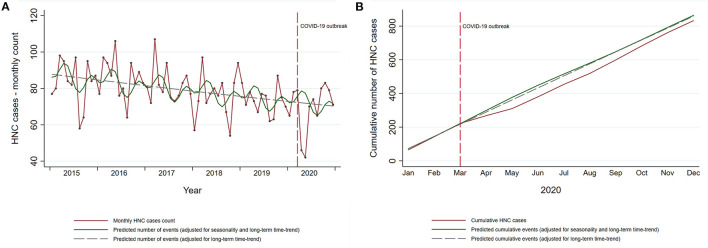
Temporal trends in incident HNC in the Piedmont Region, Italy. **(A)** Temporal trends in observed monthly HNC cases in Piedmont (red line) and the predicted number of cases estimated on the data from the pre-pandemic period (green solid and gray dashed line). **(B)** Cumulative observed and predicted HNC cases in 2020. Cumulative predicted HNC cases for 2020 are estimated on the data from the pre-pandemic period (2015–2019).

During the 5-year period before the COVID-19 outbreak (2015–2019) there were 4,811 admissions for incident HNC in Piedmont (Table 1). Apart from the year 2016, when the yearly number of patients increased by 2.9% compared with 2015, there had been a continuous annual decrease by 4.4, 7.3, and 3.4% in 2017, 2018, and 2019, respectively. During this period the incident HNC admissions decreased by 3.6% per year (95% CI: 1.6–5.6%). There were 832 admissions in 2020, of which 689 occurred after the COVID-19 outbreak in Italy (March 2020). In the months of April and May 2020, the period of the complete lockdown in Italy, there was an abrupt decline in the incident HNC admissions in Piedmont (Figure 2A). The months following June 2020 have seen a gradual increase in the number of admissions, with the cumulative number of admissions for 2020 reaching, but never surpassing the expected numbers (Figure 2B).

**Table 1:** Baseline population characteristics.

**Baseline characteristics**	**Total**	**Pre-pandemic period**	**Pandemic period**	*p*-value^*^
	**(2015–2020)**	**(January 2015–February 2020)**	**(March 2020–December 2020)**	
	***N*** **(%) or median (IQR)**			
Incident HNC cases^†^ in Piedmont	5,643	4,954	689	/
Median age, years (IQR)	68.9 (59.9–77.5)	68.9 (59.9–77.5)	68.5 (60.5–77.8)	0.864
**Sex**				
Male	4,169 (73.9)	3,655 (73.8)	514 (74.6)	0.645
Female	1,474 (26.1)	1,299 (26.2)	175 (25.4)	
**Tumor subsite**				
Oral cavity	1,435 (25.4)	1,253 (25.3)	182 (26.4)	0.403
Oropharynx	947 (16.8)	819 (16.5)	128 (18.6)	
Larynx	1,786 (31.7)	1,576 (31.8)	210 (30.5)	
Other HNC subsites	1,475 (26.1)	1,306 (26.4)	169 (24.5)	
Median length of stay, days (IQR)	2 (1–11)	2 (1–11)	3 (1–13)	0.045
**Romano/Charlson comorbidity index**				
<2	4,246 (75.2)	3,754 (75.8)	492 (71.4)	0.013
≥2	1,397 (24.8)	1,200 (24.2)	197 (28.6)	
**Primary treatment**				
Surgical	3815 (67.6)	3384 (68.3)	431 (62.6)	0.002
Non-surgical	1828 (32.4)	1570 (31.7)	258 (37.4)	
**Period**				
2015	1,001	1,001	/	/
2016	1,030	1,030	/	
2017	985	985	/	
2018	913	913	/	
2019	882	882	/	
January–February 2020	143	143	/	
March–May 2020^‡^	167	/	167	
June– September 2020^§^	289	/	289	
October–December 2020^||^	233	/	233	

*IQR, Interquartile range*.

* *Chi-square test and Wilcoxon-Mann-Whitney test, where appropriate*.

† *Patients ≥18 years of age with the following ICD-9-CM codes: 140.0–150.0, 161.0–161.9 and 195.0, and no HNC diagnosis registered in 10 years prior to the index diagnosis*.

*First pandemic wave and national lockdown in Italy*.

§ *Re-opening period and lockdown easing in Italy*.

|| *Second pandemic wave with nationally imposed restrictions in Italy*.

**Figure 3:**
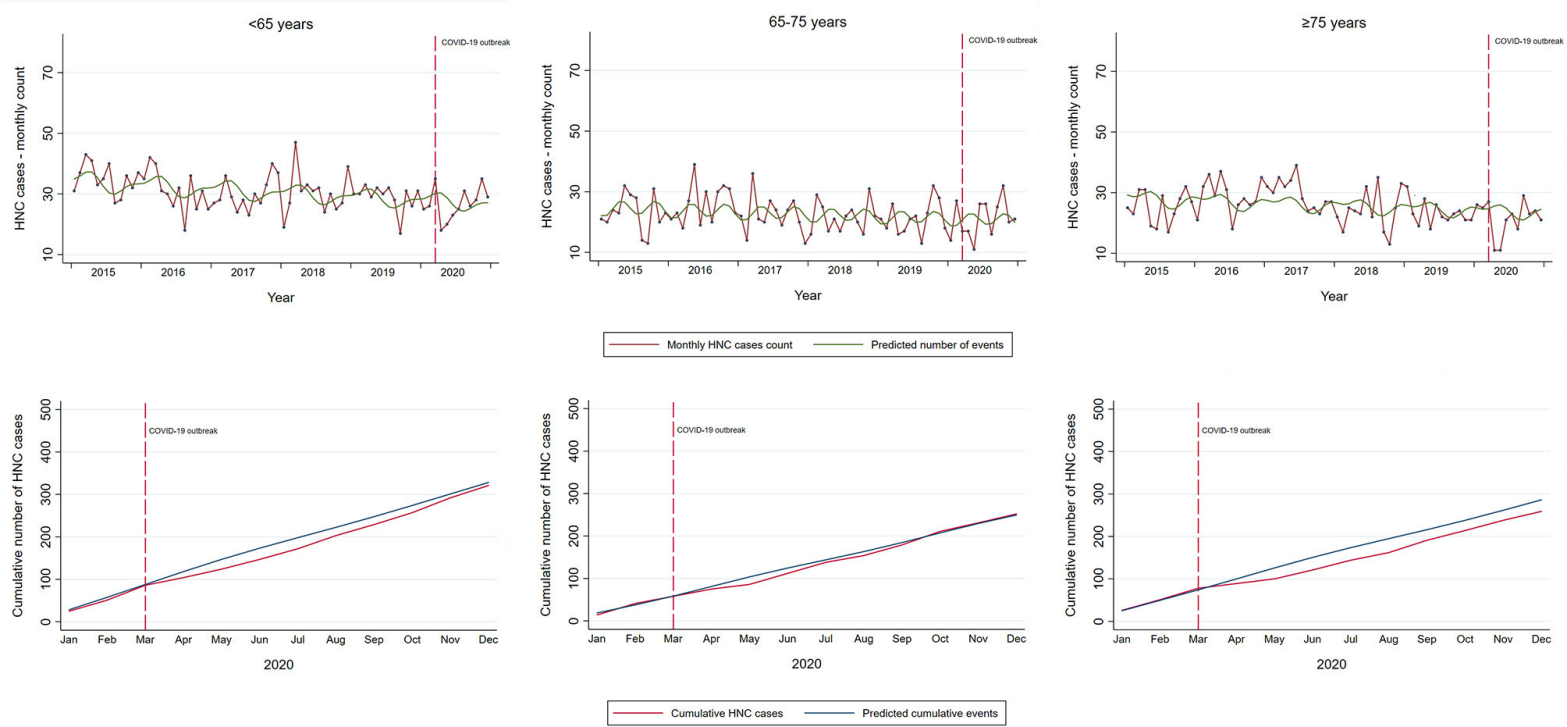
Temporal trends in incident HNC in the Piedmont Region, Italy, by age group. Temporal trends in observed monthly and cumulative HNC cases in Piedmont (red line) and the predicted number of cases estimated on the data from the pre-pandemic period with a linear term for time and Fourier terms to model holiday-related seasonality in the HNC detection (green line upper panel; blue line lower panel). Vertical red dashed line indicates the start of the COVID-19 outbreak in Italy with national restrictions.

Overall, there was no evidence of a large change in the cumulative yearly HNC admissions after the COVID-19 outbreak (RR 0.96, 95% CI: 0.89–1.03; Table 2). However, in the analysis restricted to first pandemic wave only (March–May 2020), there was a decrease of 28% in incident HNC admissions (RR 0.72, 95% CI: 0.62–0.84). Similar estimates, slightly lower in magnitude, were found in the models unadjusted for seasonal fluctuations. During the first pandemic wave, observed admissions for incident HNC were lower than expected for all age groups (Table 2). While the gap between the cumulative observed and expected totals for patients <75 years was recovered in the first 10 months of the pandemic, the admissions of patients ≥75 years remained lower than the expected by the end of 2020 (RR 0.88; 95% CI: 0.76–1.01, Table 2; Figure 3). Further stratification revealed RRs of 0.89 (95% CI: 0.75–1.04) and 0.84 (95% CI: 0.63–1.10) for patients 75–85 years and ≥85 years, respectively.

**Table 2:** Observed and expected numbers and rate ratio (RR) with 95% confidence intervals (CI) of incident HNC in the total COVID-19 period and in the first pandemic wave, specifically.

**Models**	**Total COVID-19 period**			**First pandemic wave**		
	**March 1, 2020–December 31, 2020**			**March 1, 2020–May 31, 2020**		
	**Observed**	**Expected**	**RR (95% CI)**	**Observed**	**Expected**	**RR (95% CI)**
Main model^*^	689	719	0.96 (0.89–1.03)	167	232	0.72 (0.62–0.84)
**Sensitivity analysis^†^**	689	713	0.97 (0.90–1.04)	167	216	0.77 (0.66–0.90)
**Stratified models**						
**Age group**						
<65 years	270	271	1.00 (0.88–1.12)	73	89	0.82 (0.64–1.03)
65–75 years	211	212	1.00 (0.87–1.14)	45	66	0.68 (0.50–0.91)
≥75 years	208	237	0.88 (0.76–1.01)	49	76	0.64 (0.48–0.85)
**Tumor subsite**						
Oral cavity	182	187	0.97 (0.84–1.13)	47	60	0.78 (0.58–1.04)
Oropharynx	128	134	0.96 (0.80–1.14)	25	42	0.60 (0.39–0.88)
Larynx	210	225	0.93 (0.81–1.07)	49	72	0.68 (0.50–0.90)
Other HNC sites	169	174	0.99 (0.83–1.13)	46	59	0.78 (0.57–1.04)
**Comorbidity index**						
<2	492	538	0.91 (0.84–1.00)	117	175	0.67 (0.55–0.80)
≥2	197	181	1.09 (0.94–1.25)	50	57	0.88 (0.65–1.16)
**Primary treatment**						
Surgical	431	491	0.88 (0.80–0.97)	109	160	0.68 (0.56–0.82)
Non-surgical	258	228	1.13 (1.00–1.28)	58	72	0.81 (0.61–1.04)

* *Expected events are estimated from the pre-COVID-19 period, with a linear term for time and Fourier terms to model holiday-related seasonality in the HNC detection*.

† *Expected events are estimated from the pre-COVID-19 period, with a linear term for time*.

*Romano-Charlson comorbidity index*.

Admissions for HNC subsites were similarly affected by the COVID-19 pandemic, with no clear evidence of a yearly reduction in any of the subsites in 2020 (Table 2; Supplementary Figure 1). Specifically, the first lockdown period was associated with a reduction in admissions for incident oropharyngeal (RR 0.60, 95% CI: 0.39–0.88) and laryngeal tumors (RR 0.68, 95%CI: 0.50–0.90), but with a subsequent substantial catch up by the end of the year.

Despite no evidence of the large reduction in the cumulative incident HNC admissions in 2020, there was a decline in the admission of patients with less comorbidities (CCI score <2, RR 0.91, 95% CI: 0.84–1.00), and patients undergoing primary surgical treatment (RR 0.88, 95% CI: 0.80–0.97, Table 2; Supplementary Figures S2, S3). This reduction was greater during the first pandemic wave, with a reduction of 33% (95% CI 25–45%) compared with the expected for patients with low CCI, and a reduction of 32% (95% CI 18–44%) for surgically treated patients.

### Assessment of Disease Stage at Diagnosis

**Figure 4:**
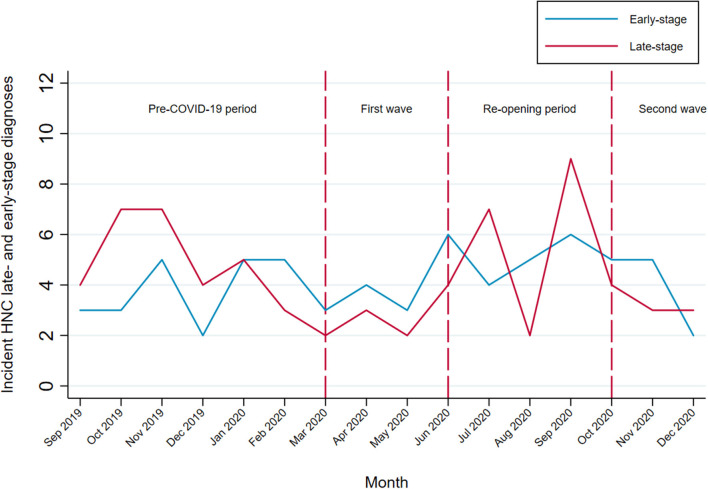
Temporal trends in HNC diagnosed at late and early stage among patients eligible for the HEADSpAcE study recruitment. Disease stage at diagnosis (TNM Classification of Malignant Tumors, AJCC cancer staging manual, VIII ed): early-stage (I and II) and late-stage (III and IV).

From September 1, 2019 to December 31, 2020, there were 135 incident HNC patients eligible for the ongoing HEADSpAcE study recruitment. Overall, approximately half of the patients were diagnosed at early and half at late stage (Table 3). Temporal trends in HNC diagnosed at late and early stage are shown in Figure 4. We observed no clear stage migration between the pre-pandemic period and the periods following the COVID-19 outbreak. The proportion of patients diagnosed at late stage in the 6 months prior to the pandemic was 56.6%, and it fell to 41.2 and 45.5% during the first (*p*-value = 0.268) and the second pandemic wave (*p*-value = 0.378), respectively, while it remained similar during the first post-COVID-19 re-opening period in summer 2020 (*p*-value = 0.595).

**Table 3:** Baseline characteristics of patients with HNC eligible for the HEADSpAcE study recruitment in the Turin center.

**Baseline characteristics**	**Period of recruitment**				
	**Total**	**Pre-COVID-19**	**First pandemic wave**	**Re-opening**	**Second pandemic wave**
		**(September 2019–February 2020)**	**(March 2020–May 2020)**	**(June 2020–September 2020)**	**(October 2020–December 2020)**
	***N*** **(%) or mean (SD)**				
**Total**	135	53	17	43	22
**Tumor subsite**					
Oral cavity	75 (55.6)	34 (64.1)	7 (41.2)	24 (55.8)	10 (45.4)
Oropharynx	27 (20.0)	7 (13.2)	3 (17.6)	11 (25.6)	6 (27.3)
Larynx	29 (21.5)	10 (18.9)	5 (29.4)	8 (18.6)	6 (27.3)
Other HNC sites	4 (2.9)	2 (3.8)	2 (11.8)	0 (0)	0 (0)
**Age at diagnosis (years)**	69.4 (12.4)	69.6 (14.8)	70.0 (10.3)	69.5 (11.6)	68.5 (9.7)
Stage at diagnosis^*^					
Early (I–II)	66 (48.9)	23 (43.4)	10 (58.8)	21 (48.8)	12 (54.6)
Late (III–IV)	69 (51.1)	30 (56.6)	7 (41.2)	22 (51.2)	10 (45.4)
*p*-value^†^	/	Reference	0.268	0.595	0.378

* *AJCC Cancer staging Manual, VIII edition*.

† *Chi-squared test*.

## Discussion

The COVID-19 pandemic has hampered healthcare systems in many countries with significant effects on the management of patients with cancer (21). Using an interrupted time-series analysis, we showed that the initial reduction of 28% in admissions for incident HNCs in the Italian region Piedmont, during the first wave of the COVID-19 pandemic, was almost completely addressed by the end of 2020. The overall trend with a sharp decline during the first pandemic wave and the subsequent increase with the cumulative numbers reaching those expected for 2020 were also observed in the analyses stratified by tumor subsites and in patients <75 years. Our findings also indicate that the pandemic has affected certain groups of patients differentially. In particular, COVID-19 caused a notable decline in diagnosis of older patients (≥75 years), patients with less comorbidities, and those treated surgically. This gap between observed and expected numbers was only partially addressed by the end of 2020, likely due to the emerging second pandemic wave in autumn 2020. During the first pandemic wave people were generally advised to stay at home and avoid presenting at their physicians and emergency departments for non-urgent conditions. It is possible that patients with generally good health status (less comorbidities) sustained ignoring initial warning cancer signs and that the created backlog in the detection of HNC in these patients will be addressed only subsequently. An observed decline in patients with primary surgical treatment can, at least partially, be explained by the reduced hospital activity and the guidelines that recommended avoidance of clinical examinations and procedures presumed to be high-risk by potentially aerosolizing the virus, such as endoscopic procedures and tracheostomy (22–24).

Delayed HNC diagnosis is generally driven by patient-related factors (low educational attainment, low socioeconomic position, fear), healthcare system factors, and disease-related factors (length of the asymptomatic period, tumor subsite, HPV status) (25–28). The crisis that COVID-19 imposed on healthcare systems and individual changes in healthcare utilization and health-seeking behaviors have likely enhanced most of the existing barriers to timely diagnosis. Moreover, the increased overall mortality due to COVID-19 that was experienced by the Piedmont population (29), especially by older and the most vulnerable individuals, such as patients with cancer, may have acted as a competing phenomenon, preventing either the diagnosis or the first hospital admission of some patients. This is further supported by our finding of a particularly strong decline in diagnosis of patients ≥75 years, which was not completely recovered by the end of 2020.

In the secondary analysis of patients eligible for recruitment in an ongoing study, there was no evidence of an increase in diagnoses at advanced stage in the periods following the first pandemic wave. This is in line with a recent finding of no increased total laryngectomy volume, reserved for advanced cases, during the COVID-19 pandemic (30). Although these findings indicate no evident stage migration, it should be considered that the 10-month period after the start of the pandemic may still be limited to observe any change in the distribution of stages at diagnosis.

Several previous studies that reported COVID-19-related reductions in HNC admissions and diagnoses (12–16, 31, 32) were hospital-based studies, conducted on a relatively limited population, focused on the first pandemic wave only, and did not account for temporal trends in HNC detection. The reported reductions in HNC referrals and diagnoses in the first pandemic months varied from 12% to up to 60% in different contexts and populations (13, 15, 16, 30, 31). In this study, accounting for temporal trends over a relatively long period and seasonal (likely holiday-related) fluctuations in HNC admissions, we found a decline of 28% in incident HNC admissions during the first pandemic wave, which was almost completely recovered in 2020. The analysis on the subgroup of patients with information on stage at diagnosis supports these findings, as we did not find evidence of a shift toward more advanced diseases at diagnosis in the months after the first pandemic wave. Our findings are based on HDRs from a population of more than 4 million inhabitants, which is one of the largest studies so far assessing the impact of COVID-19 on HNC admissions/diagnoses. These findings also demonstrate a resilience of the Piedmont healthcare system showing that the very difficult experiences of the first pandemic wave were not protracted and that there was a bounce back recovery in the management of patients with HNC.

Limitations of the current study include common flaws of studies using routinely collected administrative data, such as accuracy of data coding and limited clinical information. In particular, we had no data on the stage at diagnosis for the analysis on all incident HNC admissions in Piedmont. For this reason, we complemented the analysis with the results of a subgroup of patients undergoing an active recruitment for whom this information was available. Even if this group was relatively small, information on stage was of high quality as each patient was staged specifically for this study based on available pathological and clinical data. Another point to consider is the possible misclassification of the tumor subsites within the broader HNC category. Although we believe that our algorithm identified almost all HNC admissions, it is possible that a definite ICD-9 diagnosis was not established during the first HNC admission. Nevertheless, any such misclassification would only influence our null results of the analysis stratified by tumor subsite and would not affect the overall study findings. Finally, we believe that HNC diagnoses outside hospitals, potentially not captured by HDRs, are minor, as in case of a suspicion of HNC, general practitioners and dentists (both public and private) refer patients, almost exclusively, toward specialized hospitals, where both the biopsy and the pathologic report can be performed at one place.

## Conclusions

Here, we report the temporal trends in the incident HNCs in the first 10 months of the COVID-19 pandemic, in one of the largest Italian regions. Although an initial drop in the HNC admissions has been mostly recovered in this period, we found substantial differences in some patient subgroups. The impact of the COVID-19 on HNC observed in this study is potentially only the tip of the iceberg—the long-term effects of the pandemic on oncological outcomes and healthcare systems remain to be determined.

## Data Availability Statement

The raw data supporting the conclusions of this article will be made available by the corresponding author, upon reasonable request.

## Ethics Statement

The studies involving human participants were reviewed and approved by Ethics Committee of the University of Turin. The patients/participants of the HEADSpAcE study provided their written informed consent to participate in this study.

## Author Contributions

MP: had full access to all the data in the study, takes responsibility for the integrity of the data, the accuracy of the data analysis, and drafting of the manuscript. LR and MP: concept and design. MP, LR, VF, CM, GM, and MG: acquisition and analysis. MP, LR, and GM: statistical analysis. All authors: interpretation of data and critical revision of the manuscript for important intellectual content. All authors contributed to the article and approved the submitted version.

## Funding

This study received funding from the European Union's Horizon 2020 Research and Innovation Program under Grant Agreement No. 825771 (HEADSpAcE project), and from the Italian Ministry for Education, University and Research (Ministero dell'Istruzione, dell'Università e della Ricerca—MIUR) under the programme Dipartimenti di Eccellenza 2018–2022.

## Author Disclaimer

Where authors are identified as personnel of the International Agency for Research on Cancer/World Health Organization, the authors alone are responsible for the views expressed in this article and they do not necessarily represent the decisions, policy or views of the International Agency for Research on Cancer/World Health Organization.

## Conflict of Interest

The authors declare that the research was conducted in the absence of any commercial or financial relationships that could be construed as a potential conflict of interest.

## Publisher's Note

All claims expressed in this article are solely those of the authors and do not necessarily represent those of their affiliated organizations, or those of the publisher, the editors and the reviewers. Any product that may be evaluated in this article, or claim that may be made by its manufacturer, is not guaranteed or endorsed by the publisher.

## References

[1] OECD/European Union. How Resilient Have European Health Systems been to the COVID-19 Crisis? In Health at a Glance: Europe 2020: State of Health in the EU Cycle. Paris: OECD Publishing (2020).

[2] Blay JY Boucher S Le Vu B Cropet C Chabaud S Perol D. Delayed care for patients with newly diagnosed cancer due to COVID-19 and estimated impact on cancer mortality in France. ESMO Open. (2021) 6:100134. 10.1016/j.esmoop.2021.100134 33984676 PMC8134718

[3] Lai AG Pasea L Banerjee A. Estimated impact of the COVID-19 pandemic on cancer services and excess 1-year mortality in people with cancer and multimorbidity: near real-time data on cancer care, cancer deaths and a population-based cohort study. BMJ Open. (2020) 10:e043828. 10.1136/bmjopen-2020-043828 33203640 PMC7674020

[4] Gurney JK Millar E Dunn A Pirie R Mako M Manderson J. The impact of the COVID-19 pandemic on cancer diagnosis and service access in New Zealand-a country pursuing COVID-19 elimination. Lancet Reg Health West Pac. (2021) 10:100127. 10.1016/j.lanwpc.2021.100127 33778794 PMC7983868

[5] Ferrara G De Vincentiis L Ambrosini-Spaltro A Barbareschi M Bertolini V Contato E. Cancer diagnostic delay in Northern and Central Italy during the 2020 lockdown due to the coronavirus disease 2019 pandemic. Am J Clin Pathol. (2021) 155:64–8. 10.1093/ajcp/aqaa177 32995855 PMC7543252

[6] Maringe C Spicer J Morris M Purushotham A Nolte E Sullivan R. The impact of the COVID-19 pandemic on cancer deaths due to delays in diagnosis in England, UK: a national, population-based, modelling study. Lancet Oncol. (2020) 21:1023–34. Erratum in: *Lancet Oncol*. (2021) 22:e5. 10.1016/S1470-2045(20)30388-0 32702310 PMC7417808

[7] Baldi E Savastano S. Fear of contagion: one of the most devious enemies to fight during the COVID-19 pandemic. Disaster Med Public Health Prep. (2021) 15:e8–e9. 10.1017/dmp.2020.338 32907670 PMC7653481

[8] Schutte HW Heutink F Wellenstein DJ van den Broek GB van den Hoogen FJA Marres HAM. Impact of time to diagnosis and treatment in head and neck cancer: a systematic review. Otolaryngol Head Neck Surg. (2020) 162:446–57. 10.1177/0194599820906387 32093572

[9] Neal RD Tharmanathan P France B Din NU Cotton S Fallon-Ferguson J. Is increased time to diagnosis and treatment in symptomatic cancer associated with poorer outcomes? Systematic review. Br J Cancer. (2015) 112(Suppl. 1):S92–107. 10.1038/bjc.2015.48 25734382 PMC4385982

[10] Abrahão R Anantharaman D Gaborieau V Abedi-Ardekani B Lagiou P Lagiou A. The influence of smoking, age and stage at diagnosis on the survival after larynx, hypopharynx and oral cavity cancers in Europe: the ARCAGE study. Int J Cancer. (2018) 143:32–44. 10.1002/ijc.31294 29405297

[11] Schimansky S Lang S Beynon R Penfold C Davies A Waylen A. Association between comorbidity and survival in head and neck cancer: results from Head and Neck 5000. Head Neck. (2019) 41:1053–62. 10.1002/hed.25543 30549147 PMC6890487

[12] Tevetoglu F Kara S Aliyeva C Yildirim R Yener HM. Delayed presentation of head and neck cancer patients during COVID-19 pandemic. Eur Arch Otorhinolaryngol. (2021) 278:5081–5. 10.1007/s00405-021-06728-2 33674926 PMC7935695

[13] De Luca P Bisogno A Colacurcio V Marra P Cassandro C Camaioni A. Diagnosis and treatment delay of head and neck cancers during COVID-19 era in a tertiary care academic hospital: what should we expect? Eur Arch Otorhinolaryngol. (2022) 279:961–5. 10.1007/s00405-021-06834-1 33929609 PMC8085654

[14] Yang Y Shen C Hu C. Effect of COVID-19 epidemic on delay of diagnosis and treatment path for patients with nasopharyngeal carcinoma. Cancer Manag Res. (2020) 12:3859–64. 10.2147/CMAR.S254093 32547222 PMC7260542

[15] Kiong KL Diaz EM Gross ND Diaz EM Jr. Hanna EY. The impact of COVID-19 on head and neck cancer diagnosis and disease extent. Head Neck. (2021) 43:1890–7. 10.1002/hed.26665 33650276 PMC8013528

[16] DATA-CAN. New Data on the Impact of COVID-19 on Head and Neck Cancers. Available online at: https://www.data-can.org.uk/latest/new-data-on-the-impact-of-covid-19-on-head-and-neck-cancers (accessed August 12, 2021).

[17] HEADSpAcE. International Agency for Research on Cancer/World Health Organization. Available online at: https://headspace.iarc.fr/ (accessed September 27, 2021).

[18] Romano PS Roos LL Jollis JG. Adapting a clinical comorbidity index for use with ICD−9-CM administrative data: differing perspectives. J Clin Epidemiol. (1993) 46:1075–9. 10.1016/0895-4356(93)90103-8 8410092

[19] Presidenza del Consiglio dei, Ministri, Dipartimento della Protezione Civile. COVID-Italia. Available online at: https://github.com/pcm-dpc/COVID-19 (accessed September 1, 2022).

[20] Breslow NE Day NE. Statistical Methods in Cancer Research, Volume II: The Design and Analysis of Cohort Studies. New York, NY: Oxford University Press (1987). p. 406. 3329634

[21] Vrdoljak E Sullivan R Lawler M. Cancer and coronavirus disease 2019 how do we manage cancer optimally through a public health crisis? Eur J Cancer. (2020) 132:98–9. 10.1016/j.ejca.2020.04.001 32335477 PMC7165280

[22] Wu V Noel CW Forner D Zhang ZJ Higgins KM Enepekides DJ. Considerations for head and neck oncology practices during the coronavirus disease 2019 (COVID-19) pandemic: Wuhan and Toronto experience. Head Neck. (2020) 42:1202–8. 10.1002/hed.26205 32338807 PMC7267482

[23] Givi B Schiff BA Chinn SB Clayburgh D Iyer NG Jalisi S. Safety recommendations for evaluation and surgery of the head and neck during the COVID-19 pandemic. JAMA Otolaryngol Head Neck Surg. (2020) 146:579–84. 10.1001/jamaoto.2020.0780 32232423

[24] De Luca P Scarpa A Ralli M De Vincentiis M Cassandro E Chiarella G. Nasal, pharyngeal and laryngeal endoscopy procedures during COVID-19 pandemic: available recommendations from national and international societies. Eur Arch Otorhinolaryngol. (2020) 277:2151–3. 10.1007/s00405-020-06028-1 32377857 PMC7202461

[25] Carvalho AL Pintos J Schlecht NF Oliveira BV Fava AS Curado MP. Predictive factors for diagnosis of advanced-stage squamous cell carcinoma of the head and neck. Arch Otolaryngol Head Neck Surg. (2002) 128:313–8. 10.1001/archotol.128.3.313 11886350

[26] Nieminen M Atula T Bäck L Mäkitie A Jouhi L Aro K. Factors influencing patient and health care delays in Oropharyngeal Cancer. J of Otolaryngol Head Neck Surg. (2020) 49:22. 10.1186/s40463-020-00413-w 32326977 PMC7181590

[27] Stefanuto P Doucet JC Robertson C. Delays in treatment of oral cancer: a review of the current literature. Oral Surg Oral Med Oral Pathol Oral Radiol. (2014) 117:424–9. 10.1016/j.oooo.2013.12.407 24556495

[28] Rogers SN Pabla R McSorley A Lowe D Brown JS Vaughan ED. An assessment of deprivation as a factor in the delays in presentation, diagnosis and treatment in patients with oral and oropharyngeal squamous cell carcinoma. Oral Oncol. (2007) 43:648–55. 10.1016/j.oraloncology.2006.08.001 17070094

[29] Scortichini M Schneider Dos Santos R De' Donato F De Sario M Michelozzi P Davoli M. Excess mortality during the COVID-19 outbreak in Italy: a two-stage interrupted time-series analysis. Int J Epidemiol. (2021) 49:1909–17. 10.1093/ije/dyaa169 33053172 PMC7665549

[30] Noel CW Li Q Sutradhar R Eskander A. Total laryngectomy volume during the COVID-19 pandemic: looking for evidence of stage migration. JAMA Otolaryngol Head Neck Surg. (2021) 147:909–11. 10.1001/jamaoto.2021.2019 34410331 PMC8377599

[31] Arduino PG Conrotto D Broccoletti R. The outbreak of Novel Coronavirus disease (COVID-19) caused a worrying delay in the diagnosis of oral cancer in north-west Italy: the Turin Metropolitan Area experience. Oral Dis. (2021) 27(Suppl. 3):742–3. 10.1111/odi.13362 32306459 PMC7264792

[32] Thompson JA Lubek JE Amin N Joy R Dyalram D Ord RA. Impact of the novel coronavirus 2019 (COVID-19) pandemic on head and neck cancer care. Otolaryngol Head Neck Surg. (2022) 166:93–100. 10.1177/01945998211004544 33784206 PMC8010374

