# Supplementary material for: The Impact of the COVID-19 Pandemic on Head and Neck Cancer Diagnosis in the Piedmont Region, Italy: Interrupted Time-Series Analysis
Source: Front Public Health. 2022 Feb 21;10:809283. doi: 10.3389/fpubh.2022.809283 (PMC8899030; doi:10.3389/fpubh.2022.809283)

Supplementary Material

## Supplementary Figures

**Supplementary Figure 1.** Temporal trends in incident HNC in the Piedmont Region, Italy, by tumor site. Temporal trends in observed monthly and cumulative HNC cases in Piedmont (red line) and the predicted number of cases estimated on the data from the pre-pandemic period with a linear term for time and Fourier terms to model holiday-related seasonality in the HNC detection (green line left and blue line right). Vertical red dashed line indicates the start of the COVID-19 outbreak in Italy.


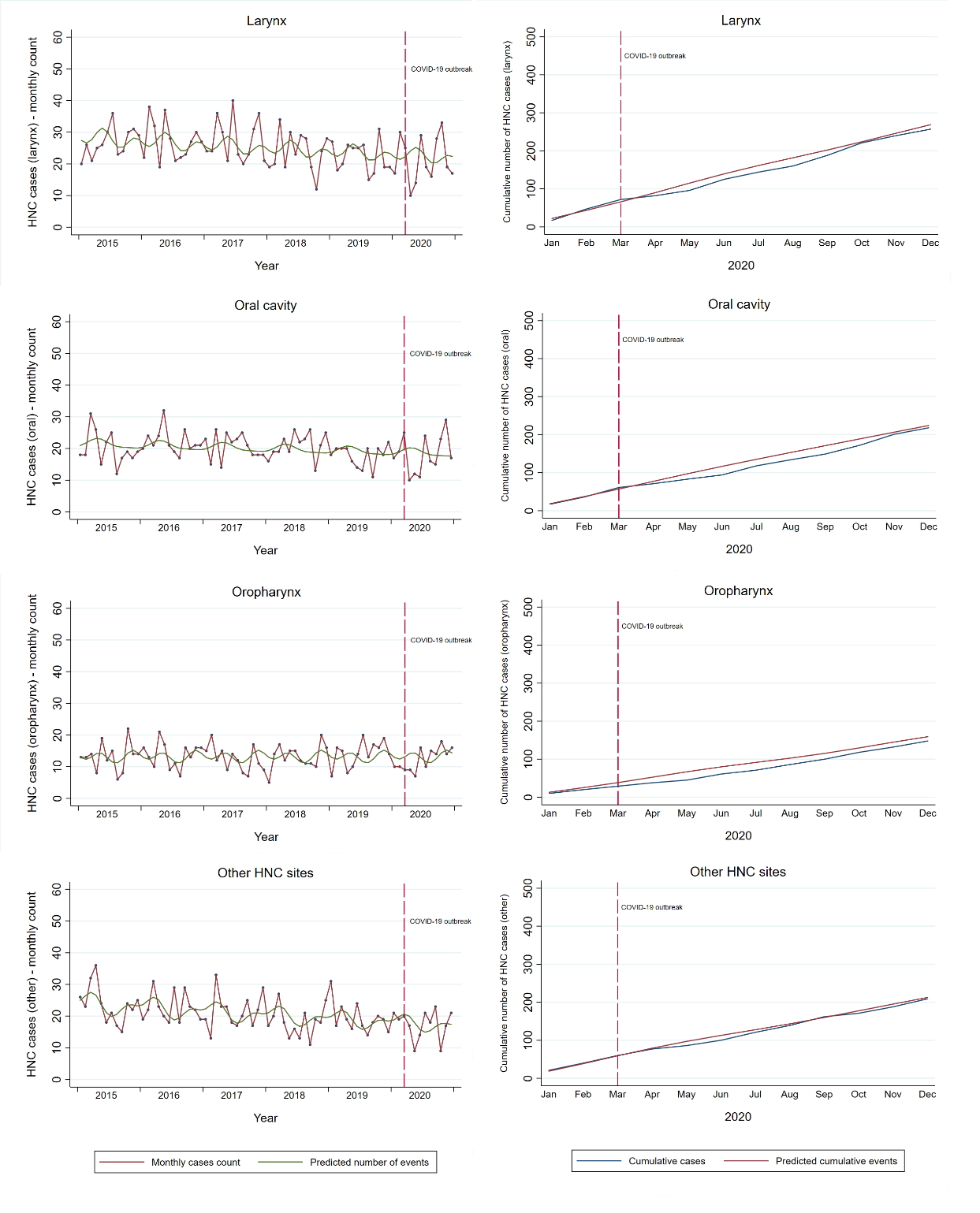


**Supplementary Figure 2.** Temporal trends in incident HNC in the Piedmont Region, Italy, by Charlson comorbidity index. Temporal trends in observed monthly and cumulative HNC cases in Piedmont (red line) and the predicted number of cases estimated on the data from the pre-pandemic period with a linear term for time and Fourier terms to model holiday-related seasonality in the HNC detection (green line upper panel; blue line lower panel). Vertical red dashed line indicates the start of the COVID-19 outbreak in Italy.


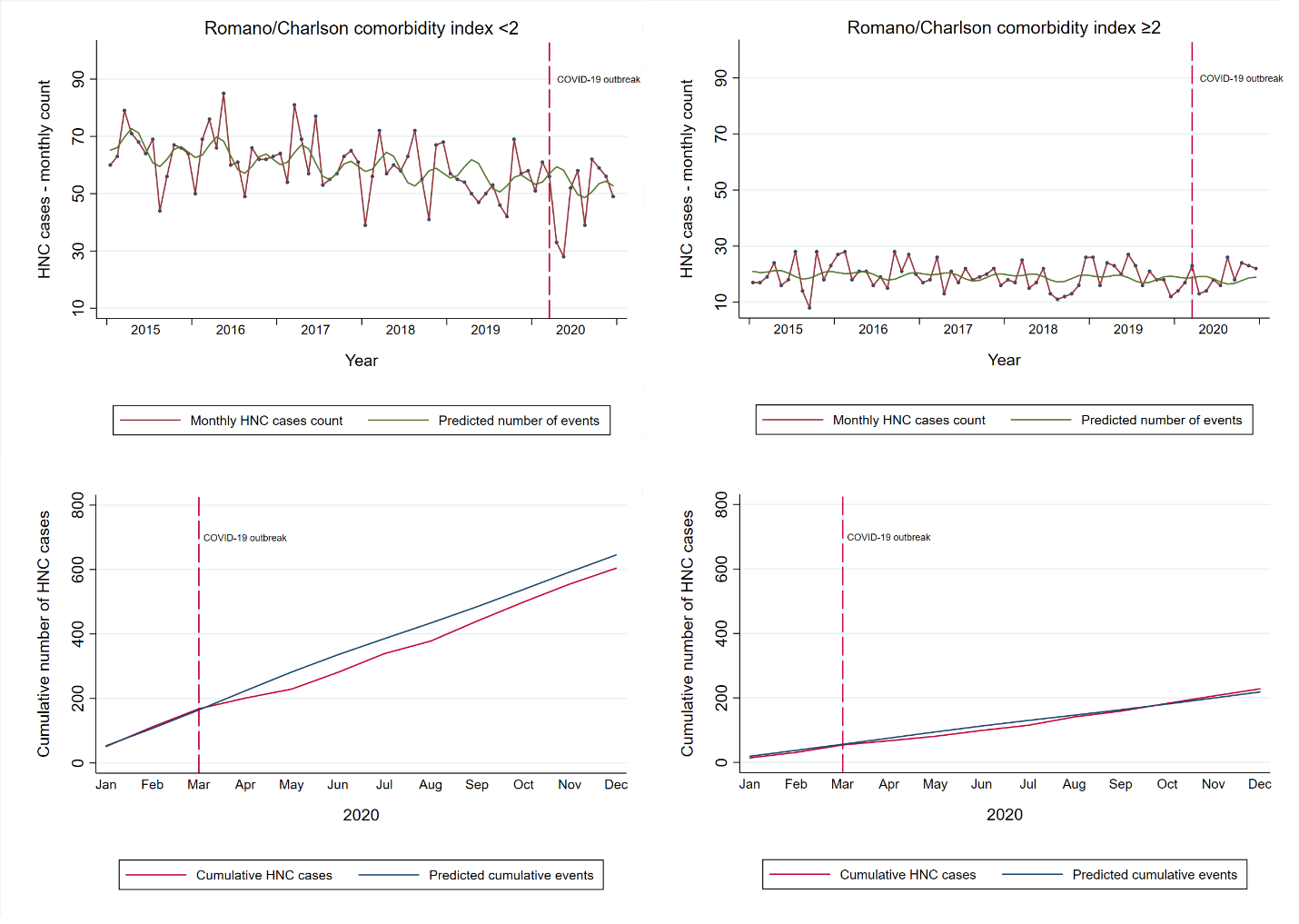


**Supplementary Figure 3.** Temporal trends in incident HNC in the Piedmont Region, Italy, by treatment within the first six months from diagnosis. Temporal trends in observed monthly HNC cases in Piedmont (red line) and the predicted number of cases estimated on the data from the pre-pandemic period with a linear term for time and Fourier terms to model holiday-related seasonality in the HNC detection (green line). Vertical red dashed line indicates the start of the COVID-19 outbreak in Italy.


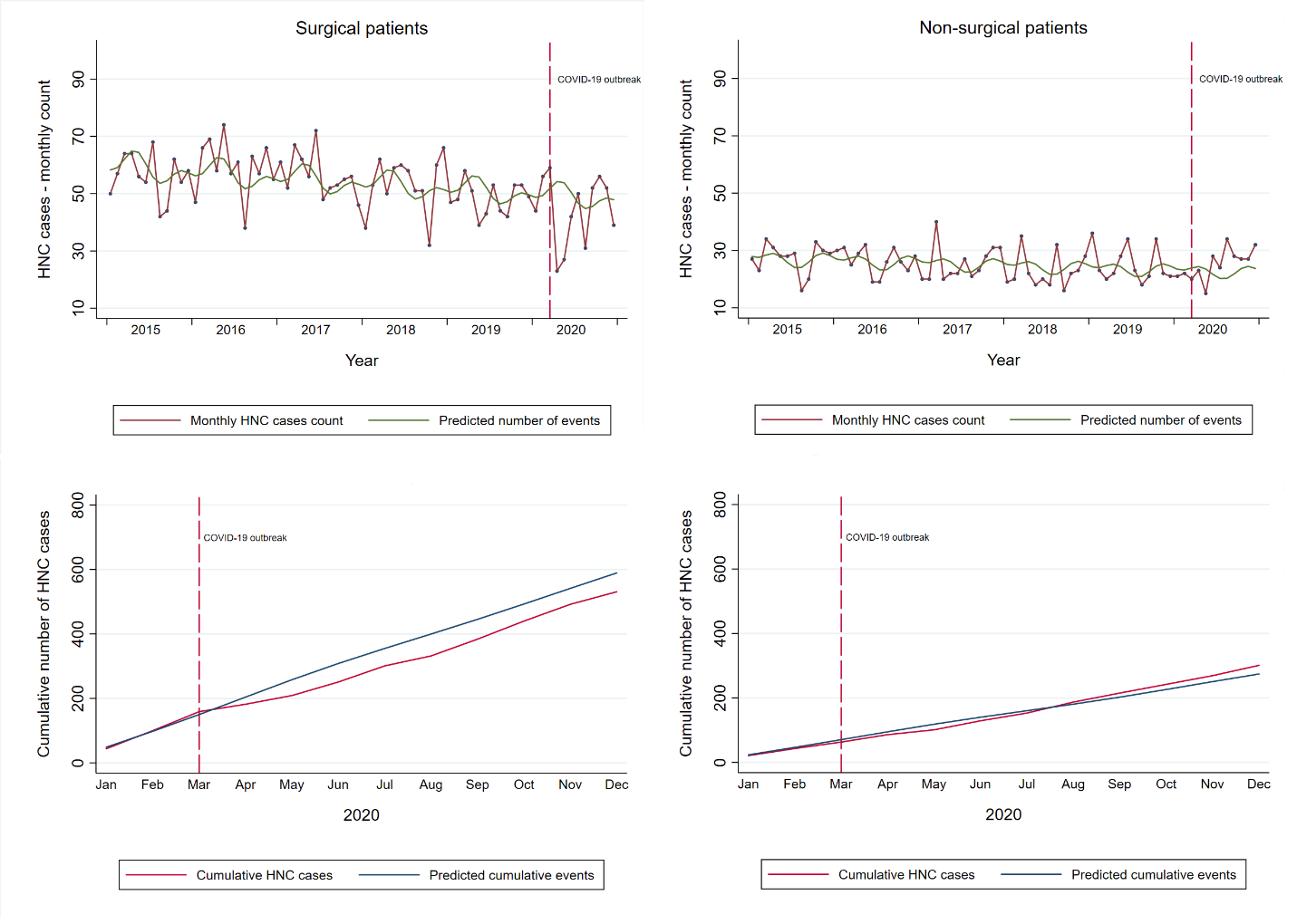

Supplement: Supplementary file 1 [file Table_1.DOCX]
